# Interference of Phenylethanoid Glycosides from *Cistanche tubulosa* with the MTT Assay

**DOI:** 10.3390/molecules20058060

**Published:** 2015-05-05

**Authors:** Yu-Jie Wang, Si-Min Zhou, Gang Xu, Yu-Qi Gao

**Affiliations:** 1 College of Ethnomedicine, Chengdu University of Traditional Chinese Medicine, Chengdu 611137, China; 2 College of High Altitude Military Medicine, Third Military Medical University, Chongqing 400038, China; E-Mails: lifengguang119@163.com (S.-M.Z.); xugangyjy@126.com (G.X.); gaoyuqiyjy@126.com (Y.-Q.G.)

**Keywords:** MTT assay, phenylethanoid glycoside, caffeic acid, cell viability, interference

## Abstract

The MTT assay, as a screening method, has been widely used to measure the viability and proliferation of cells. However, it should be noted that MTT assay may not accurately reflect the effect of *Cistanche tubulosa* ethanolic extract on EA.hy926 cells viability. To investigate and identity the components responsible for the contradictory observations of the MTT assay, echinacoside and acteoside, two main phenylethanoid glycosides, from *C. tubulosa* ethanolic extract were isolated. The data derived from CCK-8, Hoechst 33342 and annexin V-FITC/PI assays suggest that the caffeyl group present in both isolated compounds was responsible for the conflicting results of the MTT assay. These data emphasize the need of using a variety of different methods to determine the effect of medicinal agents on cell viability to avoid generating misleading results.

## 1. Introduction

The parasitic plant *Cistanche tubulosa* (Schrenk) R. Wight (*Orobanchaceae* family) is widely distributed in North African, Arab, and Asian countries [1]. The stems from *C. tubulosa* and *C. deserticola* are important in traditional Chinese medicine, having been used for the treatment of impotence, sterility, lumbago, and constipation due to colonic inertia [2]. In addition, the ethanolic extract of *C. tubulosa* has been shown to have a significant protective effect against cerebral hypoxia in mice [3].

Endothelial cells play a crucial role in the pathogenesis of hypoxic pulmonary hypertension. The EA.hy926 endothelial cell line is considerably similar to primary endothelial cells. During our own study of the anti-hypoxic effect of *C. tubulosa* ethanolic extract on EA.hy926 endothelial cell, we noticed that the 3-(4,5-dimethylthiazol-2-yl)-2,5-diphenyltetrazolium bromide (MTT) assay, which is commonly used to assess cell viability in studies of cytotoxicity and cytostatic activity, generated conflicting results by showing increased EA.hy926 cell viability after treatment.

Accurate assessment of cell viability is essential to identify potential cytotoxic or cytoprotective effects of a medicinal agent. We therefore investigated which components present in *C. tubulosa* ethanolic extract may be responsible for the conflicting results observed with the MTT assay. We isolated two main phenylethanoid glycosides (PhGs) from *C. tubulosa* ethanolic extract, echinacoside (hereafter compound **1**) and acteoside (hereafter compound **2**), and determined their effect on EA.hy926 cell viability using a variety of methods. In addition, in order to further clarify which substituent of compound **1** and **2** may be responsible for the contradictory results, we tested their aglycones, caffeic acid (hereafter compound **3**) and 3,4-dihydroxylphenylethanol (hereafter compound **4**), on EA.hy926 cell viability using the same methods.

## 2. Results and Discussion

### 2.1. Identification of Compounds

**Figure 1:**
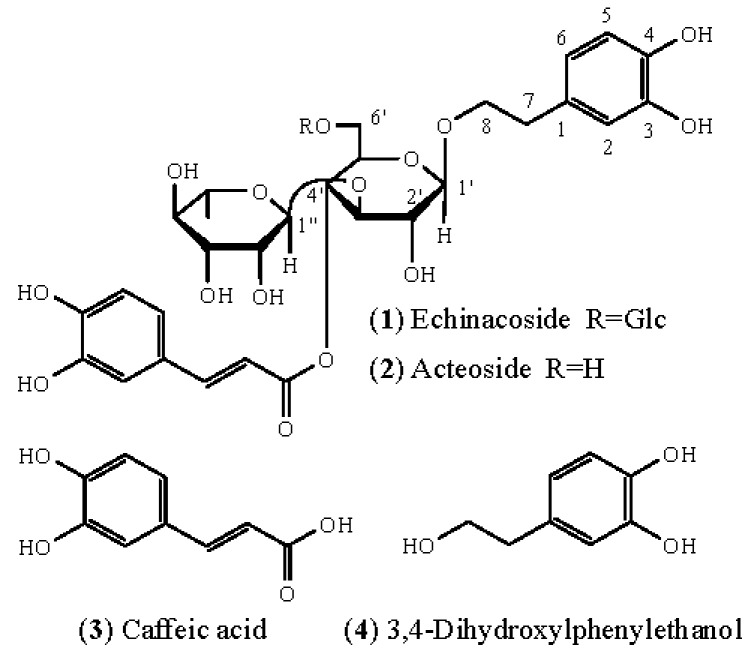
Structures of compounds **1**–**4**.

The structures of the two compounds isolated from *C. tubulosa* ethanolic extract were identified by nuclear magnetic resonance (NMR) and mass spectrometry (MS) analyses and are shown in Figure 1. Compound **1** was identified as echinacoside by comparison with previously reported NMR (Table 1) and MS (*m/z* 785 [M−H]^−^) data [4]. Compound **2** NMR spectra were highly similar to those of compound **1**, except for the absence of a β-d-glucopyranosyl group (Table 1). Compound **2** (*m/z* 623 [M−H]^−^) was identified as acteoside [5]. Importantly, both compounds have the same aglycone, which includes caffeic acid (compound **3**) and 3,4-dihydroxylphenylethanol (compound **4**).

**Table 1:** ^1^H (600 MHz) and ^13^C (150 MHz) NMR data of echinacoside (**1**) and acteoside (**2**) (CD_3_OD, δ in ppm).

Position	Echinacoside		Acteoside	
	δ_H_ (*J* in Hz)	δ_C_	δ_H_ (*J* in Hz)	δ_C_
1		130.1		130.1
2	6.69 (d, 2.0)	115.8	6.69 (d, 2.0)	115.8
3		144.7		144.7
4		143.3		143.3
5	6.67 (d, 8.0),	115.0	6.67 (d, 8.0)	115.0
6	6.57 (dd, 1.9, 8.0)	119.9	6.56 (dd, 1.9, 8.0)	119.9
7	2.79 (m, 2H)	35.2	2.79 (m, 2H)	35.2
8	3.75 (m)	70.1	3.73 (m)	70.7
	4.03 (m)		4.04 (m)	
8-*O*-Glc				
1'	4.38 (d, 7.9)	102.8	4.37 (d, 7.9)	102.8
2'	3.39 (dd, 8.2, 9.2)	74.8	3.39 (dd 7.9, 9.2)	74.8
3'	3.81 (t, 9.2)	80.3	3.81 (t, 9.2)	80.3
4'	5.00 (dd, 9.7, 9.7)	69.2	4.91 (t, 9.7, 9.8)	69.2
5'	3.71 (m)	73.4	3.59 (m)	74.6
6'	3.64 (m)	68.0	3.52 (m)	60.9
	3.93 (m)		3.61 (m)	
3'-*O*-Rha				
1''	5.18 (d, 1.8)	101.7	5.19 (d, 1.8)	101.6
2''	3.91 (dd, 1.8, 3.0)	71.0	3.92 (dd, 1.8, 3.0)	71.0
3''	3.55 (m)	70.7	3.57 (m)	70.8
4''	3.28 (m)	72.4	3.30 (m)	72.4
5''	3.53 (m)	69.1	3.55 (m)	69.1
6''	1.08 (3H, d, 6.2)	17.1	1.09 (3H, d, 6.2)	17.1
6'-*O*-Glc				
1'''	4.29 (d, 7.7)	103.3	-	-
2'''	3.19 (m)	73.7	-	-
3'''	3.54 (m)	76.4	-	-
4'''	3.26 (m)	70.8	-	-
5'''	3.23 (m)	76.5	-	-
6'''	3.62 (m)	61.2	-	-
	3.82 (m)			
4'-*O*-Caf				
1		126.3		126.3
2	7.06 (d, 2.0)	113.9	7.05 (d, 1.9)	113.9
3		145.4		145.4
4		148.4		148.4
5	6.78 (d, 8.2)	115.2	6.78 (d, 8.0)	115.2
6	6.96 (dd, 1.9, 8.2)	121.9	6.94 (dd, 1.9, 8.0)	121.8
7	7.60 (d, 15.9)	146.8	7.59 (d, 15.9)	146.6
8	6.27 (d, 15.9)	113.3	6.26 (d, 15.9)	113.4
9		167.1		166.9

### 2.2. Cell Viability Assays

**Figure 2:**
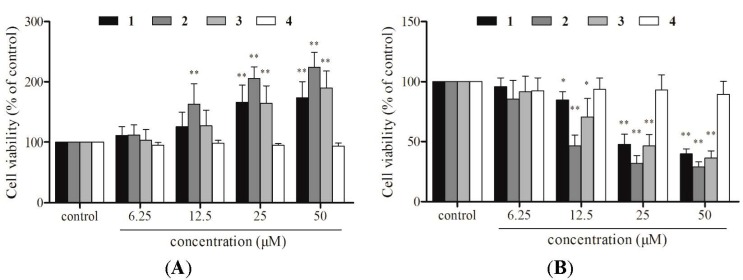
Effects of compounds **1**–**4** on cell viability. EA.hy926 cells were treated with 6.25, 12.5, 25 and 50 μM echinacoside (**1**), acteoside (**2**), caffeic acid (**3**), 3,4-dihydroxylphenylethanol (**4**) for 24 h. (**A**) MTT assay; (**B**) CCK-8 assay. Data are the mean ± S.D. of three independent experiments. * *p* < 0.05 and ** *p* < 0.01 *versus* control.

**Figure 3:**
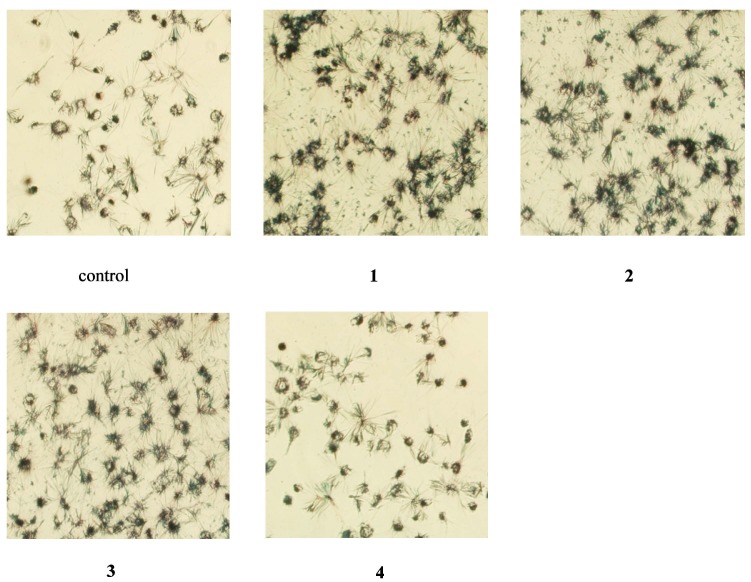
Micrographs showing formation of MTT formazan. EA.hy926 cells were treated with 50 μM echinacoside (**1**), acteoside (**2**), caffeic acid (**3**), 3,4-dihydroxylphenylethanol (**4**) for 24 h. Magnification 200×.

MTT and CCK-8 assays were used to analyze the effect of compounds **1**–**4** on EA.hy926 cell viability. The MTT assay showed that the viability of EA.hy926 cells increased significantly after treatment with 25 and 50 μM compound **1**, **2**, or **3** (166.3% and 174.1% for compound **1**, 205.8% and 224.3% for compound **2**, and 164.8% and 189.6% for compound **3**, respectively; *p* < 0.05), when compared to control cells (Figure 2A). Furthermore, morphological inspection of the cells determined that treatment with 50 μM compound **1**, **2**, or **3** increased the formation of purple formazan crystals, which are considered to be a direct indication of the proportion of living cells (Figure 3). Conversely, the CCK-8 assay suggested that cell viability decreased significantly after treatment with 12.5, 25, and 50 μM compound **1**, **2**, or **3** (75.5%, 45.1%, and 43.7% for compound **1**, 85.5%, 51.8%, and 34.3% for compound **2**, and 64.5%, 48.2%, and 36.4% for compound **3**, respectively; *p* < 0.05), compared to control cells (Figure 2B). Compound **4** did not affect cell viability in either the MTT or CCK-8 assay. The discrepancy between the results obtained with the two assays suggests that one of the two assays may not accurately reflect the effects of compounds **1** and **2** on EA.hy926 cell viability.

### 2.3. Apoptosis Assessment by Hoechst Staining

**Figure 4:**
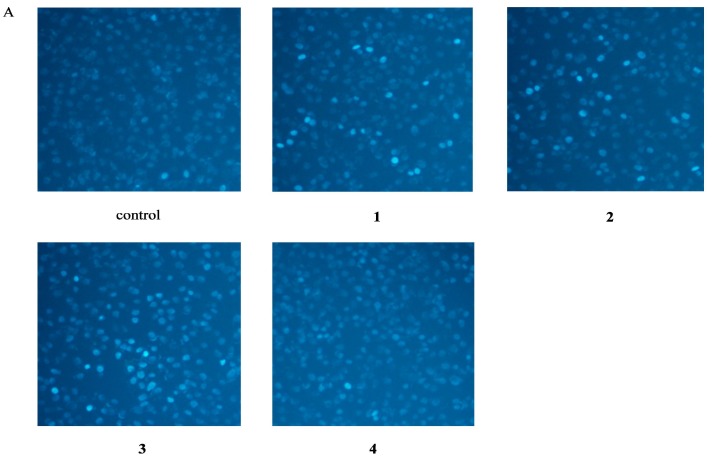

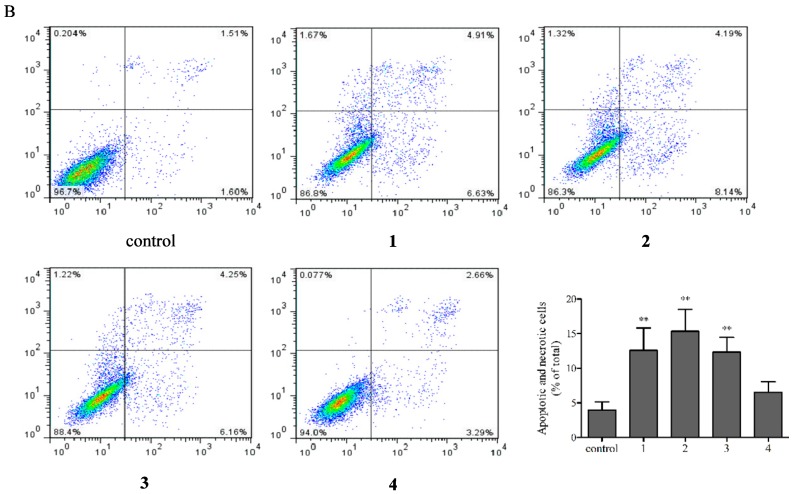
Apoptosis Assessment by Hoechst 33342 Staining and Flow Cytometry. EA.hy926 cells were treated in the absence (control), or in the presence of 50 μM echinacoside (**1**), acteoside (**2**), caffeic acid (**3**), 3,4-dihydroxylphenylethanol (**4**) for 48 h. (**A**) Apoptotic cells were identified via morphological means after staining of nuclear chromatin with Hoechst 33342 dye. Magnification 200×. (**B**) Percentage of apoptotic cells in total EA.hy926 cells. Data are the mean ± S.D. of three independent experiments. ** *p* < 0.01 *versus* control.

Hoechst 33342 is a cell-permeable DNA stain that is excited by ultraviolet light and emits blue fluorescence at 460 to 490 nm. The condensed chromatin of apoptotic cells stain more brightly than the chromatin of normal cells [6]. Whereas control cells exhibited uniformly dispersed chromatin, normal organelles, and intact cell membranes, cells incubated with 50 μM compounds **1**, **2**, or **3** for 48 h showed the typical staining of apoptotic cells (Figure 4A). Conversely, exposure of cells to 50 μM of compound **4** did not increase the number of apoptotic nuclei compared to control treatment. 

### 2.4. Flow Cytometric Analysis of Apoptotic Cells

The annexin V-FITC/PI double-staining assay identifies apoptotic cells by the high affinity binding of annexin V-FITC to phosphatidylserine, which is externalized in cells undergoing apoptosis [7]. In this assay, viable cells do not bind to either annexin V nor PI and are therefore not stained (lower left quadrant), early apoptotic cells are stained green by the binding of annexin V-FITC (lower right quadrant), and late apoptotic cells are stained green and red by the binding of annexin V-FITC to phosphatidylserine and PI to necrotic cells, respectively (upper right quadrant). As shown in Figure 4B, cells incubated with 50 μM compounds **1**, **2**, or **3** for 48 h, the percentage of apoptotic cells increased from 3.74% (control cells) to 10.32%, 12.95%, and 11.30%, respectively. Compared to control cells, compound **4** did not increase the percentage of apoptotic EA.hy926 cells.

Consistent with the results obtained with the CCK-8 assay and by Hoechst 33342 staining, the flow cytometry assay showed that compounds **1**, **2**, and **3** induced apoptosis in EA.hy926 cells. All together, these observations suggest that the increase in EA.hy926 cell viability observed with the MTT assay after treatment with compounds **1**–**3** represented a false-positive result.

### 2.5. Discussion

In 1983, Mosmann developed a colorimetric MTT microplate assay for measuring cell proliferation and cytotoxicity [8]. This simple assay, and modifications of it, is now extensively used in cell biology laboratories around the world. Fractionation studies in mammalian cells indicated that the reduced pyridine nucleotide cofactor, NADH, is responsible for most MTT reduction. This was supported by studies using whole cells [9]. MTT reduction is therefore associated with metabolically active cells and is not only coupled with mitochondrial respiration, but also with the cytoplasm and non-mitochondrial membranes including the endosome/lysosome compartment and the plasma membrane [10]. The net positive charge on the MTT molecule is the primary factor for its cellular uptake via the plasma membrane potential.

Notably, the MTT assay may occasionally not accurately reflect the actual effect that a tested compound has on specific cells. However, the chemical structures or groups that may cause conflicting results in the MTT assay have not been identified. Recent studies have demonstrated that the MTT assay could underestimate of the anti-proliferative effect of (−)-epigallocatechin-3-gallate, the most abundant polyphenol in green tea [11]. In studies using the MTT assay to detect tumor chemosensitivity, anti-cancer chemotherapeutic agents, such as epirubicine, paclitaxel, doxetaxel, and imatinib mesylate (gleevec), showed increased cell viability [12,13]. Moreover, a number of studies have reported that certain plant extracts and redox-active polyphenols could interfere with the MTT assay as they directly reduce the MTT tetrazolium salt in the absence of cells [14].

The need to solubilize the MTT formazan crystals prior to spectrophotometric analysis in a microplate reader and the inherent endpoint nature of the reaction limit the use of the MTT assay to certain applications. This led to the development of tetrazolium analogues in which the phenyl moieties were decorated with negatively charged sulfonate groups, such as 2,3-bis-(2-methoxy-4-nitro-5-sulfophenyl)-2H-tetrazolium-5-carboxanilide (XTT), a negatively charged inner salt [15] and 3-(4,5-dimethylthiazol-2-yl)-5-(3-carboxymethoxyphenyl)-2-(4-sulfophenyl)-2H-tetrazolium (MTS), a weakly acidic inner salt closely related to MTT [16]. These modifications resulted in the production of culture medium-soluble formazan products that eliminated the requirement of a solubilization step prior to quantitation. Correspondingly, as the increased negative charge on these molecules reduced their ability to move across cell membranes [17], intermediate electron acceptors (IEA), such as 1-methoxy-5-methyl phenazinium methylsulphate (1-methoxy PMS) were required to facilitate tetrazole dye reduction or to enhance the rate of reduction.

More recently, a new generation of water-soluble tetrazolium salts has been developed of which WST-1 is the prototype [18]. WST-1, a negatively charged disulfonated inner salt containing an iodine residue, is more stable than XTT and MTS in the presence of 1-methoxy PMS, its obligatory IEA. This led to the marketing of WST-1/1-methoxy PMS as a convenient single reagent cell proliferation kit. Several other tetrazolium salts in the WST series have been developed, the most useful of which perhaps being WST-8 [19]. WST-8 appears to have highly similar cellular reduction properties to WST-1 and is being marketed independently as the CCK-8 assay. WST-8 is cell impermeable and is therefore reduced extracellularly, via electron transport from intracellular NADH to WST-8 across the plasma membrane mediated by 1-methoxy PMS. Although both MTT and WST-8/1-methoxy PMS reduction are driven by intracellular NADH, the source of NADH appears to differ within these two methods. WST-8/1-methoxy PMS reduction is more highly dependent on the malate/aspartate shuttle that links mitochondrial tricarboxylic acid cycle-NADH with the extramitochondrial space [20].

In preliminary experiments, we confirmed that compounds **1**, **2**, **3**, and **4** could not directly reduce the MTT tetrazolium salt (not shown). In the present study, the direct influence of the compounds themselves with the reagent were eliminated by carefully washing the microplates with phosphate buffered saline (PBS) before MTT or CCK-8 solution was added, as indicated in the manufacturer’s protocols. Nevertheless, the results obtained using the two methods were still contradictory (Figure 2). As expected, incubation of cells with compounds **1**, **2**, and **3** increased the reduction of the tetrazolium salt into purple formazan, compared with the control group (Figure 3), suggesting that mere reduction of the tetrazolium salt in the presence of a specific compound is not sufficient to judge the compound’s ability to interfere with the MTT assay.

To determine whether compounds **1**, **2**, **3**, and **4** could induce apoptosis in EA.hy926 cells, cellular morphological changes and phosphatidylserine externalization were assessed. Consistently with the decrease in cellular viability observed with the CCK-8 assay, the results of Hoechst staining and flow cytometry analysis using annexin V-FITC/PI suggested that the growth inhibition observed after compound **1**, **2**, and **3** treatment was due, at least in part, to EA.hy926 cell apoptosis. Our results also supported the idea that the caffeyl group present in both compounds **1** and **2** was responsible for the contradictory results obtained with the MTT assay.

Similar to our findings, Rottlerin, a potent large conductance potassium channel opener, did not exhibit reactivity toward MTT *in vitro*. However, it strongly enhanced the formation of formazan crystals inside cells, a result that was in evident conflict with Rottlerin inhibition of NF-κB and cell proliferation observed by analysis of [^3^H]-thymidine incorporation into DNA [21]. The mechanism by which Rottlerin enhanced MTT reduction was attributed to its mitochondrial uncoupling effect [22]. Rottlerin has a cinnamoyl acid substituent group. Compared with cinnamoyl acid, compound **3** possesses two additional hydroxyl residues, which are located at C-3 and C-4. Therefore, we speculate that the mechanism by which compounds **1** and **2** cause conflicting results in the MTT assay may also be associated with their mitochondrial uncoupling effects. To test this hypothesis, comparative studies among compounds **1**, **2**, and a chemical uncoupler, such as trifluorocarbonylcyanide phenylhydrazone (FCCP), are needed.

## 3. Experimental Section

### 3.1. Reagents and Chemicals

Compound **3** (caffeic acid–powder, purity = 98.6%) was purchased from Chengdu Push Bio-Technology Co., Ltd. (Chengdu, China). Compound **4** (3,4-dihydroxylphenylethanol–oil, purity = 98.5%) was obtained from Chengdu Must Bio-Technology Co., Ltd. (Chengdu, China). MTT and dimethyl sulfoxide (DMSO) were obtained from Sigma-Aldrich (St. Louis, MO, USA). The CCK-8 assay was purchased from Dojin Laboratories (Kumamoto, Japan). Hoechst 33342 and annexin V-FITC/PI apoptosis detection kits were purchased from the Beyotime Institute of Biotechnology (Haimen, China).

### 3.2. Plant Material

*C. tubulosa* stems were collected from Yutian Prefecture, Xinjiang Uygur Autonomous Region, China. Voucher specimens were deposited in the Key Laboratory of High Altitude Medicine, Third Military Medical University (Chongqing, China) and authenticated by Yi Zhang from Chengdu University of Traditional Chinese Medicine (Chengdu, China).

### 3.3. Extraction and Isolation of Plant Material

Air-dried *C. tubulosa* (0.6 kg) stems were powdered and extracted with 50% ethanol for 2 h at 70 °C. After removal of the solvent under reduced pressure, the ethanolic extract (212.5 g) was dissolved and suspended in water (2 L) and extracted with *n*-butanol (2 L × 3). The *n*-butanolic extract (110 g) was chromatographed on a polyamide column and eluted with ethanol/water (0:100, 5:95, 15:85, 30:70, 50:50) producing five fractions (A–E). Fraction B (65 g) was fractionated on an ODS column; elution with ethanol:water (1:9) separated compound **1** (3 g). Fraction D (10.5 g) was fractionated on an ODS column; elution with ethanol/water (1:4) separated compound **2** (1 g).

NMR spectra were recorded on a Bruker Avance 600 spectrometer in CD_3_OD with Tetramethylsilane (TMS) as internal standard. Mass spectra were carried out on a BioTOF-Q mass spectrometer.

### 3.4. Cell Culture

EA.hy926 cell lines were obtained from the American Type Culture Collection (Manassas, VA, USA). Cells were cultured in RPMI 1640 medium, supplemented with 10% (v/v) fetal bovine serum and 1% (v/v) penicillin-streptomycin in a humidified environment with 5% CO_2_ at 37 °C.

### 3.5. Cell Viability

Cell viability was assessed using the MTT and CCK-8 assays. Compounds **1** and **2**, and their aglycones, caffeic acid (**3**) and 3,4-dihydroxylphenylethanol (**4**) were dissolved in DMSO to the final DMSO concentration < 0.1% (v/v) in culture media.

EA.hy926 cells were seeded on 96-well plates at 4 × 10^3^ cells/well. After incubation for 24 h, cells were treated with 6.25–50 μM compound **1**, **2**, **3**, or **4** for 24 h. Culture medium was removed and cells were washed twice with phosphate-buffered saline (PBS). Aliquots (10 μL) of stock MTT solution (5 mg·mL^−1^) were added to each well containing 100 μL of medium and incubated with cells for 4 h. After incubation, the medium was removed and 150 μL aliquots of DMSO were added to each well to solubilize the formazan crystals. Absorbance was measured at 490 nm using a microplate reader (Bio-Tek Synergy HT, Winooski, VT, USA). Cell viability was expressed as the percentage of MTT reduction, assigning the 100% value to the absorbance of the control cells. All experiments were performed three times and presented as mean ± standard deviation (SD).

The CCK-8 assay was performed following the literature with slight modifications [23]. Specifically, cells were treated with 6.25–50 μM compounds **1**, **2**, **3**, or **4** for 24 h and washed twice with PBS before the addition of 10 μL CCK-8 solution and 100 μL of culture media. After incubating the microplate at 37 °C for 2 h, absorbance was measured at 450 nm using a microplate reader. Cell viability was expressed as the percentage of viable cells relative to control cells (100%). All experiments were undertaken three times and expressed as mean ± SD.

### 3.6. Apoptosis Assessment by Hoechst 33342 Staining

Apoptotic morphological changes were observed by Hoechst 33342 staining. Briefly, EA.hy926 cells were seeded on 48-well plates (2 × 10^4^ cells/well) and treated with 50 μM compound **1**, **2**, **3**, or **4** for 48 h at 37 °C, washed twice with PBS, and fixed with 4% (v/v) paraformaldehyde for 15 min at room temperature. After rinsing twice with PBS, cells were stained with Hoechst 33342 (10 μg·mL^−1^) for 5 min at room temperature, and washed twice with PBS. Hoechst-stained nuclei were visualized using a fluorescence microscope (Olympus, Tokyo, Japan).

### 3.7. Apoptosis Analysis by Flow Cytometry

Apoptotic cells were detected by annexin V-FITC/PI double-staining and flow cytometry analysis. Briefly, after treatment with 50 μM compound **1**, **2**, **3**, or **4** for 48 h, cells were harvested and resuspended in PBS at 1 × 10^5^ cells·mL^−1^. After centrifugation at 500× *g* for 5 min at 25 °C, 195 μL of annexin V-FITC binding buffer and 5 μL of annexin V-FITC were added. After gentle vortex-mixing, the mixture was incubated for 10 min at room temperature in the dark. After centrifugation at 500× *g* for 5 min at 25 °C, 190 μL of FITC-conjugated annexin V binding buffer and 10 μL PI were added. After gentle vortex-mixing, samples were analyzed using a FACSCalibur Flow Cytometer (Becton Dickinson, San Jose, CA, USA) within 30 min.

### 3.8. Statistical Analysis

All experimental data were expressed as mean ± SD. The significance of the differences between experimental samples and the corresponding control was assessed by one-way analysis of variance (ANOVA) using the SPSS software (version 11.5). Statistical significance was set at *p* < 0.05.

## 4. Conclusions

Our results suggest that overestimation of the number of viable cells observed the MTT assay could mask the cytotoxicity of certain compounds. Therefore, during drug screening processes, we recommend using a variety of methods to determine the effect of the specific compound in cell viability (such as CCK-8 and ^3^H-TdR assays) to avoid generating misleading results.
